# Stereotactic body radiotherapy for pancreatic cancer – A systematic review of prospective data

**DOI:** 10.1016/j.ctro.2024.100738

**Published:** 2024-01-28

**Authors:** Mohamed A Shouman, Frederik Fuchs, Franziska Walter, Stefanie Corradini, C Benedikt Westphalen, Marlies Vornhülz, Georg Beyer, Dorian Andrade, Claus Belka, Maximilian Niyazi, Paul Rogowski

**Affiliations:** a Department of Radiation Oncology, University Hospital LMU, Munich, Germany; b Bavarian Cancer Research Center (BZKF), Munich, Germany; c Department of Medicine III and Comprehensive Cancer Center (CCC Munich LMU), University Hospital LMU, Munich, Germany; d Department of Internal Medicine II, LMU University Hospital, Munich, Germany; e Department of General, Visceral, and Transplant Surgery, University Hospital LMU, Munich, Germany; f German Cancer Consortium (DKTK), Partner Site Munich, Munich, Germany; g Department of Radiation Oncology, University Hospital Tübingen, Tübingen, Germany; h German Cancer Consortium (DKTK), Partner Site Tübingen, Germany

**Keywords:** Pancreatic cancer, PDAC, Radiotherapy, SBRT, LAPC, BRPC, local recurrence, MRgRT, SMART

## Abstract

**Purpose:**

This systematic review aims to comprehensively summarize the current prospective evidence regarding Stereotactic Body Radiotherapy (SBRT) in various clinical contexts for pancreatic cancer including its use as neoadjuvant therapy for borderline resectable pancreatic cancer (BRPC), induction therapy for locally advanced pancreatic cancer (LAPC), salvage therapy for isolated local recurrence (ILR), adjuvant therapy after radical resection, and as a palliative treatment. Special attention is given to the application of magnetic resonance-guided radiotherapy (MRgRT).

**Methods:**

Following PRISMA guidelines, a systematic review of the Medline database via PubMed was conducted focusing on prospective studies published within the past decade. Data were extracted concerning study characteristics, outcome measures, toxicity profiles, SBRT dosage and fractionation regimens, as well as additional systemic therapies.

**Results and conclusion:**

31 studies with in total 1,571 patients were included in this review encompassing 14 studies for LAPC, 9 for neoadjuvant treatment, 2 for adjuvant treatment, 2 for ILR, with an additional 4 studies evaluating MRgRT. In LAPC, SBRT demonstrates encouraging results, characterized by favorable local control rates. Several studies even report conversion to resectable disease with substantial resection rates reaching 39%. The adoption of MRgRT may provide a solution to the challenge to deliver ablative doses while minimizing severe toxicities. In BRPC, select prospective studies combining preoperative ablative-dose SBRT with modern induction systemic therapies have achieved remarkable resection rates of up to 80%. MRgRT also holds potential in this context. Adjuvant SBRT does not appear to confer relevant advantages over chemotherapy. While prospective data for SBRT in ILR and for palliative pain relief are limited, they corroborate positive findings from retrospective studies.

## Highlights

• Review of 31 prospective SBRT studies for PDAC including 1,571 patients in various clinical scenarios.
• LAPC: SBRT in LAPC shows favorable local control, potential for resection (up to 39%).
• BRPC: SBRT plus modern systemic therapies achieve high resection rates (up to 80%).
• MRgRT offers potential for optimal dosing with fewer side effects.
• Urgent need for broader randomized trials with well-defined patient cohorts to further clarify SBRT's role in PDAC.

## 1 Introduction

Pancreatic ductal adenocarcinoma (PDAC) is an extremely aggressive disease, with a 5-year overall survival (OS) rate of only approximately 12 % for all stages combined [1]. Despite representing a small proportion (3 %) of all cancer cases, pancreatic cancer ranks as the third highest cause of cancer-related deaths in the United States and in the European Union regardless of advancements in treatment options [2], [3].

The approach to treating localized PDAC is largely based on its resectability status, which depends on the tumor’s relationship to the adjacent vascular structures. According to the current NCCN guidelines, resectable disease (RPC) is characterized by the absence of tumor contact with the celiac artery, the superior mesenteric artery, or the common hepatic artery and ≤180° contact with the porto-mesenteric vein with no contour irregularity [4]. LAPC is characterized as >180° contact with arterial structures, or unreconstructible involvement of the porto-mesenteric vein, thereby defining unresectable disease [5]. Tumors that are neither clearly resectable nor unresectable are classified as BRPC. However, the definition of LAPC / BRPC is still controversial among different societies [5], [6], [7], [8], [9], [10]. In 2017, the International Association of Pancreatology aimed to enhance the accuracy of the borderline resectability definition by incorporating additional biological (CA 19–9 levels, lymph node involvement) and conditional criteria (poor performance status) [11].

The role of radiotherapy in the treatment of PDAC has been debated for the last 40 years and is still under investigation. Chemoradiotherapy (CRT) delivered daily for 5–6 weeks is still the most common treatment course [12]. However, recent phase III randomized trials have reported conflicting results for CRT demonstrating minimal or no impact on OS in BRPC and LAPC, despite improvements in local control (LC) and achieving negative margin resections [13], [14], [15].

The reason why a benefit in LC does not translate into a survival benefit is probably multifactorial and largely influenced by the high frequency of metastases observed in this disease. However, despite advancements in aggressive and effective chemotherapeutic regimens such as FOLFIRINOX and gemcitabine plus nab-paclitaxel the rate of local progression remains high[16], [17].

In response, academic centers are shifting towards new radiotherapy techniques like SBRT that targets the primary tumor with minimal margin and high single doses in few fractions. Theoretical advantages over conventional radiation include: a shorter treatment time, more focused treatment fields, a higher biological effective dose (BED), and the possibility to better spare adjacent organs at risk (OAR). These benefits are even more pronounced when the latest technology such as MR-Linac is used for SBRT. However, although there are several retrospective comparisons in favor of SBRT [18], [19], [20], [21], prospective high-level evidence is currently just being generated. Nonetheless, despite the absence of level I evidence, several guidelines have included SBRT as a viable treatment option for the respective clinical situations [22].

This systematic review aims to provide a comprehensive overview of the current prospective evidence for SBRT in the treatment of PDAC. We will highlight the role of SBRT in each clinical scenario, i.e., as induction therapy for LAPC, as neoadjuvant therapy for BRPC/RPC, as an adjuvant therapy for patients with resected pancreatic cancer, as salvage therapy for ILR, and as palliative treatment. A focus will be placed on magnetic resonance-guided radiotherapy.

## 2 Methods

A systematic review was performed in accordance with the Preferred Reporting Items for Systematic Reviews and Meta-Analyses (PRISMA) guidelines [23]. The Medline database through PubMed was systematically searched in July 2023 for prospective trials investigating oncological outcomes and toxicity of SBRT for pancreatic cancer.

Inclusion criteria were:

(1) prospective studies investigating SBRT for pancreatic cancer,
(2) published between 2013 and 2023 and
(3) publication in English language.

The specific search term utilized is reported in the appendix.

**Fig. 1:**
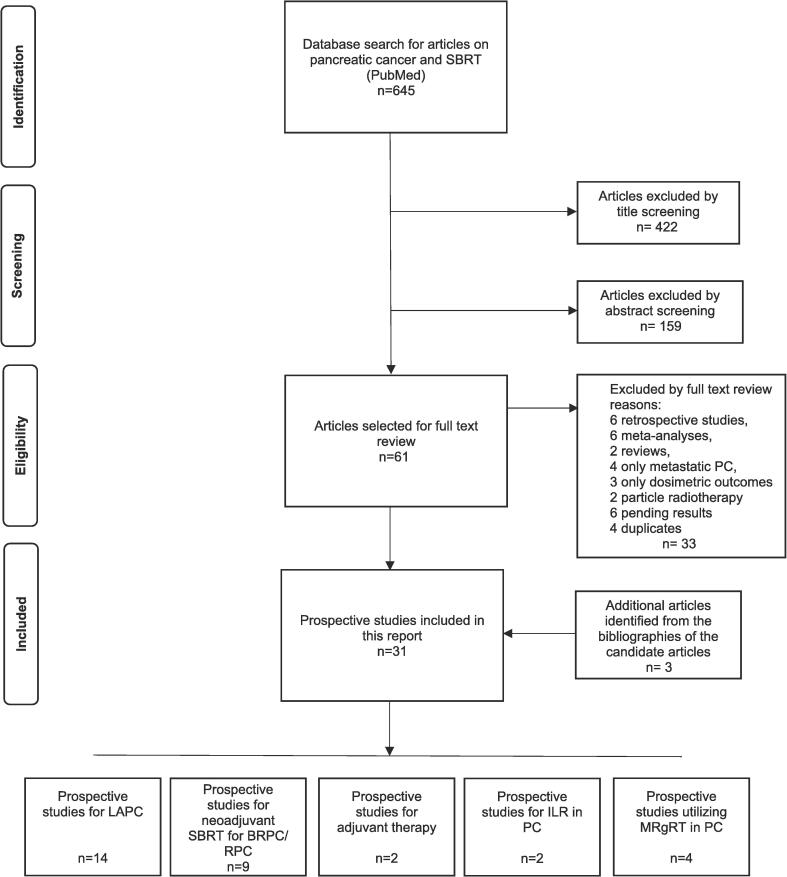
PRISMA flow diagram of the screening and selection process.

Study selection was performed in two steps. First, titles and abstracts were screened; then, selected full-text articles were screened for inclusion. Two independent investigators (MS, PR) were responsible for the eligibility screening and disagreements were handled by consensus. Additional references were identified from the bibliographies of candidate articles. The study selection process is shown in Fig. 1.

The data extraction included: first author, year of publication, study population, number of patients, experimental and control arm therapy details, median OS, median progression-free survival (PFS), LC, resection rate, as well as toxicity rates.

The included studies are discussed separately according to the different clinical scenarios. Studies that included patients from more than one clinical scenario were discussed in the appropriate section, depending on the proportion of patients. In accordance with NCCN guidelines, we referred in this review to the preoperative treatment for patients with upfront RPC or BRPC as neoadjuvant therapy. Induction therapy was used to describe the perioperative treatment for patients with LAPC [4].

## 3 Results

A total of 645 records were retrieved from the database using the search strategy. Initially, 61 studies met the criteria in the first screening phase. The second screening phase involved a thorough assessment of full texts, leading to an exclusion of 33 additional studies due to factors such as retrospective approach, *meta*-analyses, focus solely on metastatic disease, focus only on dosimetric outcomes, and utilization of particle radiotherapy. Additional 3 references were identified from the bibliographies of candidate articles. Ultimately, 31 studies with in total 1,571 patients were included. The study selection process is shown in Fig. 1.

### SBRT in patients with LAPC

LAPC accounts for 35–40 % of cases upon initial diagnosis [24]. Primary treatment aims in these patients are lengthening survival and optimizing quality of life. The optimal treatment and particularly the role of radiotherapy are controversial. Extrapolated from advances in metastatic pancreatic cancer, current guidelines recommend systemic chemotherapy with modified FOLFIRINOX or a combination of gemcitabine and nab-paclitaxel [5], [7]. In the setting of LAPC and more effective systemic treatment options, the role of local tumor control through the addition of CRT has been investigated in several studies.

In this setting, the most relevant phase III trial LAP07 failed to show an OS-benefit with the addition of CRT (54 Gy in 30 fractions with concurrent gemcitabine) compared to induction chemotherapy alone, despite being associated with an improvement in LC and a longer treatment-free interval [13]. Likewise, early findings from an interim analysis on the German CONKO-007 trial investigating the addition of gemcitabine-based CRT (50.4 Gy in 28 fractions) to induction chemotherapy with FOLFIRINOX did not show an improvement in OS [14].

These results of CRT, coupled with technical advances in radiation oncology, spurred increased exploration of SBRT in LAPC due to its benefits: shorter treatment time, less interruption of chemotherapy and dose escalation to the target volume, while minimizing exposure to nearby OAR [25]. A recent *meta*-analysis by Tchelebi et al. comparing conventional fractionated radiotherapy with SBRT in LAPC patients supports this approach suggesting an improved 2-year OS (27 % versus 14 %) and a more favorable acute toxicity profile (6 % versus 38 %) [21].

However, although early studies evaluating SBRT in 1–3 fractions demonstrated promising rates of LC, significant severe late gastrointestinal toxicity was observed [26], [27], [28]. In contrast, modern SBRT concepts for LAPC typically use 5 fractions to find a balance between high LC and acceptable toxicity.

Our search identified 14 prospective studies, consisting of one observational study, seven phase I and six phase II studies (see Table 1). Patient numbers ranged from eleven to 69. Fractionation ranged from 25 Gy to 50 Gy in 3–6 fractions. While in studies published until 2017 gemcitabine-based induction chemotherapies were common, FOLFIRINOX prevailed in the newer studies.

**Table 1:** Prospective studies using SBRT for treatment of LAPC.

Study, Year	Study design	Patients	Median SBRT Dose	Fractions	Chemotherapy	Median OS	Median PFS	Local failure (FFLP/LC)	Resection rate	Toxicity
Gurka et al. 2013 [37]	Phase I	10	25 Gy	5	Concurrent GEM (80 % 6 cycles)	12 mos. (from start date of CTX)	7 mos. (from start date of CTX)	60 % at mFu of 1-year	0 %	No G ≥ 3 RTX-related
Tozzi et al. 2013 [38]	Phase I	31 (22LAPC/9 ILR)	36 to 45 Gy (83 % 45 Gy, 17 % 36 Gy)	6	Induction GEM-based CTX 100 %: (33 % GEM only, 37 % GEMOX, 23 % GEM-5FU, 7 % PEF-G)	11 mos. (from SBRT)	8 mos. (from SBRT)	2-year FFLP 75 % (96 % at 45 Gy)	0 %	No G ≥ 3 RTX-related
Herman et al. 2015 [33]	Phase II	49	33 Gy	5	Induction CTX GEM (90 %)	14 mos. (from Dx)	8 mos. (from Dx)	1-year FFLP: 78 %	8 %	2 % G ≥ 2 acute GI, 11 % late GI
Comito et al. 2017 [34]	Phase II	45	45 Gy	6	Induction CTX (71 %) (GEMOX 17 (38 %), GEM 7 (16 %), PEF-G 6 (13 %), Altro (4 %))	13 mos. (from SBRT),19 mos. (from Dx)	8 mos. (from SBRT)	2-year FFLP: 90 %	7 %	No G ≥ 3 RTX-related
Kim et al. 2019 [40]	Observational	27	25 to 42 Gy (37 % 25 Gy/548 % 30 Gy/5, 11 % 36 Gy/3 4 % 42 Gy/3F)	3 to 5	Concurrent CTX Capecitabine (81 %)	12 mos. (form SBRT)	NR	1- year LC: 67 %	NR	22 % G 2, 22 % G 3, 0 % G 4
Liauw et al. 2020 [35]	Phase I/II	15	30 to 45 Gy (20 % 30 Gy, 20 % 37.5 Gy, 60 % 45 Gy)	3	Induction CTX 100 % (80 % FOLFIRINOX, 13 % GEM, 7 % combination of both)	13 mos. (form SBRT)	7 mos. (from SBRT)	1-year FFLP: 80 %	0 %	53 % G 2 GI 27 % G 3 + GI bleeding
Simoni et al. 2021 [45]	Phase I	59 (32 LAPC/27 BRPC)	50 Gy	5	Induction CTX 100 % (FOLFIRINOX 64 %, GEM/nab-paclitaxel 36 %)	All: 10 mos. (from SBRT) 30 mos. (from Dx)	All: 11 mos. (from SBRT)19 mos. (from Dx) , Resected:21 mos. (from Dx) 14 (from SBRT) Non-resected:14 mos. (from Dx) 6 mos. (from SBRT)	1-year FFLP: Resected: 85 % Non resected79.7 % 2-year FFLP: Resected: 80 % Non resected 60.6 %	BRPC: 89 %, LAPC: 34 % (57.7 % R0)	No G ≥ 3 RTX-related
Qing et al. 2021 [41]	Phase I	16	35 to 45 Gy (25 % 35 Gy, 18.75 % 37.5 Gy, 18.75 % 40 Gy, 18.75 % 42.5 Gy, 18.75 % 45 Gy)	5	Adjuvant CTX (87 %) after SBRT: (44 % S1, 31 % Gemcitabine, 123 % combination of both)	15 mos. (From SBRT)	10 mos. (from SBRT)	median LPFS: 13 mos.	0 %	31 % G1-2 of acute GI No G 3 or 4 GI toxicities, (3 hematologic toxicities and 1 biliary)
Zhu et al. 2021 [42]	Phase II	63	35 to 37 Gy (Median Dose 36.0 Gy)	5	Sequential S-1 CTX	14 mos. (From SBRT)	10 mos. (from SBRT)	NR	0 %	14 % G ≥ 3 acute GI, 5 % late GI
Teriaca et al. 2021 LAPC-1 trial[36]	Phase II	39	40 Gy	5	Induction FOLFIRINOX: median 8 cycles (2–8)	18 mos. (From start date of CTx)	11 mos. (NR)	1-year LC rate: 81 %, 3-year LC rate: 53 %	18 %	10 % G ≥ 3 toxicity
Courtney PT et al. 2021 [43]	Phase I	30 (19 LAPC/3 mPC/ 8 unresectable)	40 to 50 Gy: (11 % 40 Gy, 53 % 45 Gy, 36 % 50 Gy)	5	Induction CTx in 20 patients (67 %): 30 % FOLFIRINOX, 40 % GEM/ nab-paclitaxel, 3 % Gemcitabine alone, 4 % other Adjuvant CTx in 10 patients: 33 %	All: 17 mos. (from Dx),10 mos. (from SBRT)LAPC: 19 mos. (from Dx, 12 mos. from SBRT)	NR	cumulative incidence at 1-year: 14 %	0 %	23 % G ≤ 2 acute toxicity, 7 % G 4 to 5 late toxicity (at 45 Gy)
Hill et.al. 2022 [46], [48]	Phase II	48 (44 LAPC/ 4ILR)	33 Gy	5	Modified FOLFIRINOX (mFFX), or GEM and nab-paclitaxel (GnP)	22 mos. (from Dx) 15 mos. (from SBRT)	13 mos. (from Dx)6 mos. (from SBRT)	LPFS:24 mos. (from Dx) ,16 mos. (from SBRT)	39 %, (75 % R0)	2.1 % late grade ≥ 2 GI
van 't Land et al. 2023 LAPC-2 trial[48]	phase I/II	38	40 Gy	5	Induction mFOLFIRINOX median 8 cycles/ six bi-weekly intradermal vaccinations with IMM-101 (92 %).	19 mos. (From start of CTX)	12 mos. (from start of CTx)	LPFS: 15 mos.	21 % (75 % R0)	34 % G 3, no G 4 3 % G 5 none related to IMM-101.
Reyngold et al. 2023 [49]	Phase I	24	27 to 33 Gy (37.5 % 27 Gy, 33 % 30 Gy, 29 % 33 Gy)	3	Induction CTx for a median of 4 mos.: 63 %received mFOLFIRINOX, 25 % received Gemcitabine/nab-paclitaxel	24 mos. (for patients with CA19-9 ≤ 60U/mL), 11 mos. (for patients with CA19-9 > 60U/mL)	2-year PFS: 21 %	2- year LC: 32 %	16 %	No G ≥ 3 toxicities

**Abbreviations:** BRPC: borderline resectable pancreatic cancer; CTx: Chemotherapy; Dx: Diagnosis; DLT: Dose limiting toxicity; FOLFIRINOX: oxaliplatin + folinic acid + irinotecan + fluorouracil; G: Grade; FFLP: Freedom from local progression; GI: Gastrointestinal; GEM: Gemcitabine; GEMOX: Gemcitabine-Oxaliplatin; Gy: Gray; LAPC: Locally advanced pancreatic cancer; LC: Local control; LPFS: Local progression-free survival; mFu: median Follow-up; mos.: months; N/A = not applicable; NR: not reported; OS: Overall survival; PEF-G: Cisplatinum-Epirubicin-Fluorouracil-Gemcitabine; PFS: Progression-free survival; resect: resected; R0: negative resection; RT: Radiotherapy; SBRT: Stereotactic body radiotherapy.

In general, the median OS in the included studies consistently ranged between twelve and 24 months, thus confirming data from single-institution retrospective series and a large, pooled analysis of mainly retrospective series [29], [30], [31]. Nonetheless, the contribution of SBRT to survival remains unclear. In a phase II study treating LAPC patients solely with FOLFIRINOX median survival was ten months [32]. However, inter-study comparisons are not recommended and randomized trials are warranted to investigate a survival benefit of the addition of SBRT to current standard induction chemotherapy. Several of the included studies in our analysis reported LC rates between 78 % and 90 % at one year [33], [34], [35], [36]. This is in line with a pooled LC of 72 % in the analysis of Petrelli [31]. Of note, their study found that a higher total dose and a greater number of treatment fractions were linked to improved LC.

Nevertheless, for selected LAPC cases, the aim of induction therapy may also be to achieve tumour downsizing to allow resection [7]. Regarding the conversion rate to resectable disease, studies included in our analysis reported variable results. While in nine studies no patient underwent surgery or data was not reported [35], [37], [38], [39], [40], [41], [42], [43], [44], the resection rate ranged from 7 % to 39 % in the other nine studies [33], [34], [36], [45], [46], [47], [48], [49], [50]. There are several reasons for this variability: First, there are different definitions of unresectable disease in current guidelines [5], [6], [51], [52] resulting in patients with a tumor stage close to “borderline resectable” and patients with “never resectable” disease categorized the same. Second, surgery in LAPC patients who respond favorably to neoadjuvant therapy often involve complex reconstructions due to the proximity of these tumors to arterial and venous vessels requiring trained surgeons operating in high-volume centers. Therefore, it is possible that resection was excluded in some studies ab initio. Third, the choice of chemotherapy influences the resection rate [53]. This can be observed in our analysis, since all studies achieving a resection rate ≥ 18 % used multi-agent chemotherapy [36], [46], [48], [54].

Regarding safety of SBRT, rates of ≥ grade 3 toxicity ranged between 0 % and 34 %, which is comparable with the grade 3 and 4 toxicity rate of 0 % to 36 % in the pooled analysis of Petrelli [31]. In five studies, the ≥ grade 3 toxicity rate was 0 %. Even in combination with multi-agent chemotherapy (specifically FOLFIRINOX or gemcitabine + nab-paclitaxel) the safety and effectiveness of SBRT was affirmed in a phase II trial by Hill [46]. The study showed a median OS of 15 months from SBRT and a resection rate of 39 % (75 % R0). The median OS in resected patients was 22 months from SBRT. Only one patient (2 %) experienced late ≥ grade 2 gastrointestinal toxicity due to SBRT.

However, two studies included in our analysis reported grade 4 and grade 5 toxicities potentially attributable to radiotherapy [48], [43]. This must be seen against the background that in the natural course of LAPC disease, luminal organs and vessels are frequently infiltrated by the tumor, which itself leads to substantial morbidity. In addition, the patients in the respective studies were treated multimodally, so that a clear attribution of events to radiation was not possible.

Nevertheless, this underlines the fine line between keeping the dose low to OAR and escalating the dose to the tumor. The latter is important since 25 – 33 Gy in 5 fractions as used in several of the included prospective studies corresponds to a BED with an α/β of 10 (BED_10_) of < 55 Gy, which is lower than in the CRT-fractionations of 50.4 Gy to 54 Gy (BED_10_ ∼ 60 to 64 Gy) and well below the ablative doses sought with SBRT [55]. However, sole conversion in BED fails to consider factors such as acceleration and uncertainties regarding the true α/β ratio of PDAC [56], [57]. Nevertheless, there is retrospective evidence that escalating radiation dose to a BED_10_ > 70 Gy resulted in improved median OS (18 versus 15 months) and freedom from local progression (FFLP) (10 versus 6 months) compared to the standard CRT dose of 54 Gy in 30 fractions [58]. This was achieved with a slightly hypofractionated concept of 57.25 Gy in 25 fractions. Hypofractionated doses were even increased in another retrospective study reaching a BED_10_ ∼ 98 Gy in 15 and 25 fractions demonstrating a favorable 2-year OS of 38 % in combination with a relatively low rate of grade 3 toxicity (13 %) [59]. In general, employing a hypofractionated, ablative approach with 12–15 fractions appears intriguing. This is due to the high α/β ratio in PDAC and the low α/β ratios in adjacent organs, rendering them vulnerable to high doses.

Dose escalation to a BED_10_ of 70 Gy to 100 Gy can be achieved by a simultaneous integrated boost (SIB) technique allowing the dose to be increased in a defined boost volume, while other target volumes (i.e. areas with risk for microscopic spread) get covered by a lower and safer dose [60], [61]. Several studies have demonstrated promising results in the effectiveness and feasibility of this SIB strategy, despite variations in dose and fractionation protocols [30], [62]. To further mitigate the risk of severe late toxicity arising from high doses in the adjacent OAR, a simultaneous integrated protection (SIP) technique has been introduced by Brunner [63]. This approach involves creating an additional safety margin around the OARs defined as the planning organ at risk volume (PRV), which is subsequently subtracted from the target volume in a second step. The combination of SIB with SIP offers another opportunity to widen the therapeutic window, thereby enhancing the overall efficacy of the treatment for LAPC.

Another strategy to improve the efficacy of SBRT is the combination with molecularly targeted agents. Lin conducted a prospective phase I dose escalation study combining nelfinavir (an HIV protease inhibitor and AKT inhibitor) with SBRT in patients with BRPC and LAPC [54]. The treatment was well tolerated at the highest dose level of nelfinavir (1250 mg twice daily) combined with SBRT to 40 Gy in 5 fractions. The rate of ≥ grade 3 gastrointestinal bleeding was 11 %. Results showed a median OS of 14 months, along with an excellent LC rate of 85 % at one year. The efficacy of combining immunotherapy with radiation in the treatment of LAPC is currently under investigation. Although there are no published studies that specifically demonstrate the advantages of using immunotherapy for LAPC, research in this area is ongoing. In a recent phase I/II trial, the safety of combining SBRT with IMM-101 (heat-killed mycobacterium) for patients with non-progressive LAPC after mFOLFIRINOX was assessed [48]. The combination proved to be safe as there were no significant adverse events related to IMM-101. However, incorporating heat-killed mycobacterium IMM-101 into SBRT did not show any improvement in progression-free survival 12 months with a median OS of 19 months.

A more technological approach to escalate dose in the tumor without compromising OAR is the use of MRgRT, which will be discussed in the following section.

### 3.2 MR Linac-Based radiotherapy

PDAC has a low radiosensitivity and emerging evidence indicates that increasing the prescribed radiation dose to an ablative level can enhance LC and possibly OS [59], [64], [65], [66]. However, most of the prospective studies on 5-fraction RT for PDAC have used nonablative radiation doses due to the proximity of gastrointestinal luminal OAR and concerns about potential severe adverse effects. One promising method to enhance the radiation dose while complying with constraints on nearby critical organs is through the utilization of advanced image guidance techniques, such as magnetic resonance-guided radiation therapy (MRgRT). MR-Linac systems combine an onboard MRI unit with a linear accelerator (1) providing improved imaging of soft tissues compared to standard radiotherapy CT imaging, (2) enabling daily interfractional online-adaptive treatment planning and (3) offering real time visualization and intrafractional monitoring of the target using continuous cine MR image [67], [68], [69]. An alternative term frequently used is “Stereotactic MR-guided Adaptive Radiotherapy” (SMART). MRgRT / SMART, respectively, can lead to a more precise target volume and OAR definition allowing for smaller PTV margins and eventually a dose escalation while respecting all dose constraints. Several dosimetric analyses have shown the efficacy of this approach in terms of tumor coverage and OAR sparing [70], [71], [72]. In addition, MR imaging does not expose patients to additional ionizing radiation and eliminates the need for invasive fiducial marker implantation.

Our search identified four prospective trials on MRgRT published between 2018 and 2023 consisting of two observational studies, one phase I and one phase II trial treating mixed patient collectives with predominantly LAPC (Table 2).

**Table 2:** Prospective studies using MR guided SBRT for the treatment pancreatic cancer.

Study, Year	Study Phase	Patients	SBRT Dose (Gy)	Fractions	CTx	Intervention	Median OS (Mos.)	Local Control (LC)	Toxicity	Resection rate
Heerkens et al. 2018 [39]	Phase II	20 (18 LAPC, 2 medically inoperable /refused surgery)	24 Gy	3	No induction or concurrent CTX, 4 patients received CTX after SBRT	24 G in 3 fractions in 1 week.	9 mos. (from SBRT)	NR	No G3/G4	NR
Bordeau et al. 2022 [47]	Observational	70 (63 PDAC) (49 LAPC, 3 BRPC, 1 RPC, 4 mPC, 6 ILR, 1 medically inoperable, 6 mPC & ILR)	50 Gy (range 30–50)	5	Induction CTx (FOLFIRINOX 56 %, GEM-ABRAXANE 2 %, FOLFOX 10 %, GEMCITABINE 3 %, FOLFIRI 2 %)	50 Gy in 5 consecutive fractions.	21 mos. (from SBRT) 22 mos. (from CTX)	1-yr.:87 % 2-yr.:81 %	2 % acute G3, 4 % late G3	LAPC: 39 % (100 % R0)
Doppenberg et al. 2023 [44]	Observational	74 LAPC	40 Gy (range 32–40 Gy)	5	Induction FOLFIRINOX 88 %, Gemcitabine 12 %	40 Gy in 5 fractions within two weeks	12 mos. (from SBRT) 20 mos. (from Dx)	1-yr.: 91 %	3 % acute G ≥ 3, late G 3 %	NR
Parikh et al. 2023 [50]	Phase II	136 (77 LAPC, 59 BRPC)	50 Gy	5	Induction CTx (mFOLFIRINOX 65 % or gemcitabine/nab-paclitaxel 17 %)	50 Gy in 5 consecutive fractions.	1-year OS 65 %	1-yr.: 83 %	7 % ≥3 G3 GI toxicity	All: 32 % BRPC: 56 % LAPC: 14 %

**Abbreviations:** BRPC: borderline resectable pancreatic cancer; CTx: Chemotherapy; Dx: Diagnosis; FOLFIRINOX: oxaliplatin + folinic acid + irinotecan + fluorouracil; FOLFIRI: Leucovorin + irinotecan + fluorouracil; FOLFOX: Leucovorin + irinotecan + Fluorouracil, G = Grade; FFLP: Freedom from local progression; GI: Gastrointestinal; Gy: Gray; LAPC: Locally advanced pancreatic cancer; LC: Local control; LPFS: Local progression-free survival; mPC: metastatic pancreatic cancer; RPC: resectable pancreatic cancer; NR: not reported; OS: Overall survival; PDAC: Pancreatic ductal adenocarcinoma; PFS: Progression-free survival; R0: negative resection margin; RT: Radiotherapy; SBRT: Stereotactic body radiotherapy; SMART: stereotactic MR-guided adaptive radiation therapy.

The safety and technical feasibility of MRgRT was shown by Heerkens et al. in a phase I study, which found no cases of grade 3 acute or late toxicity. However, dose was 24 Gy in 3 fractions corresponding to a rather low BED_10_ of 43.2 Gy [39].

Doppenberg reported results of an observational study of 74 LAPC patients treated with MRgRT of 40 Gy delivered in 5 fractions (BED_10_ = 72 Gy) after induction chemotherapy (mFOLFIRINOX in 88 %). They observed a median OS of 20 months from diagnosis and 12 months from the start of SBRT and a one-year LC rate of 90 %, which is in line with series on a conventional linac. However, toxicity was mild with only 3 % grade 3 acute and late toxicity, respectively [44].

Another prospective observational study was presented by Bordeau. The study included 70 predominantly LAPC patients who received a radiation dose of 50 Gy in 5 fractions. The majority of patients (86 %) had induction chemotherapy prior to MRgRT. The reported median OS was 21 months and LC at one and two years were 87 % and 81 %. Of note, 39 % of LAPC patients were eventually resected with a 100 % R0 rate. Toxicity was very low [47], [73].

Furthermore, an international phase II study investigated MRgRT with 50 Gy in 5 fractions after induction chemotherapy in 136 patients diagnosed with LAPC or BRPC. The 1-year OS was 94 % from diagnosis and 65 % from MRgRT, while the 1-year LC was 83 %. The resection rates among the BRPC and LAPC patients were 56 % and 14 %. Toxicity was acceptable with 9 % possibly related acute ≥ 3 grade gastrointestinal toxicity [50].

A forthcoming phase III trial, known as LAP-ABLATE, will investigate the efficacy of induction chemotherapy with or without MRgRT with 50 Gy in 5 fractions for treating LAPC in a group of 267 patients [74].

In summary, MRgRT for PDAC shows benefits in terms of tumor coverage and OAR sparing, is feasible without limiting toxicity and reaches promising OS and LC rates. It has the potential to become the future gold standard for treating patients with LAPC. However, the implementation of adaptive techniques also requires additional time and resources, as plans need to be reoptimized between treatment sessions. This makes MRgRT costly and resource-intensive, since it involves multidisciplinary teams to re-contour images and review and approve adapted plans on a daily basis [68], [69].

### 3.3 SBRT in patients with BRPC/RPC

Surgical resection is considered the primary curative approach in PDAC, however, only 15–20 % of all newly diagnosed cases are clearly resectable [75], [76]. Another 15 % are classified as BRPC at diagnosis, depending on the definition used [5], [7]. Approximately 35 % to 60 % of patients undergoing surgery have positive margins, and this rate tends to be higher in patients with BRPC [77], [78]. The 5-year survival rate drops from around 25 % to about 10 % in individuals who have positive surgical margins [79], [80]. This is underscored by the fact that 50 % to 86 % of PDAC patients experience local recurrence following margin-positive resection [81], [82]. In turn, there is evidence that when an initially unresectable finding is converted to an operable finding after neoadjuvant treatment with R0 resection, survival rates are similar to those achieved for up-front RPC [83].

These facts provide a strong rationale for preoperative treatment with the aims of tumor downstaging and eradicating microscopic tumor in order to increase the R0 resection rate. Moreover, neoadjuvant therapy may help identify patients with unfavorable tumor biology, in whom disease progresses early, and spare them futile resection with potentially significant morbidity.

However, no consensus exists regarding the optimal neoadjuvant strategy. Multi-agent chemotherapy regimens like FOLFIRINOX and gemcitabine / nab-paclitaxel have shown efficacy and tolerability, but the role of radiotherapy remains controversial [84].

Two recent randomized trials have shown that neoadjuvant CRT may provide benefits compared to upfront surgery in patients with BRPC. The first study, conducted by Jang, was terminated early due to improved OS with neoadjuvant versus adjuvant CRT with 54 Gy in 30 fraction and concomitant gemcitabine (21 months versus 12 months). The resection rate was higher in the neoadjuvant group with 78 % versus 63 % in the upfront surgery group, with a higher rate of R0 resections (52 % versus 26 %) [85].

The phase III PREOPANC −1 trial showed that neoadjuvant gemcitabine followed by CRT (36 Gy in 15 fractions) improved the R0 resection rate (71 % versus 40 %), disease-free survival, and FFLP in patients with RPC and BRPC compared to up-front surgery [86]. However, there was no significant improvement in OS in the long-term results (16 versus 14 months) [15]. A pre-defined subset analysis revealed an OS benefit specifically in BRPC patients but not in RPC patients.

Another ongoing trial, PREOPANC-2, aims to answer the question of which neoadjuvant strategy is most effective - chemotherapy or CRT. 368 RPC or BRPC patients will be randomized between 8 cycles of preoperative FOLFIRINOX, and preoperative CRT (PREOPANC-1 regimen) followed by surgery and 4 cycles of adjuvant gemcitabine [87]. Results are eagerly awaited.

Although the “traditional” focus of research on pancreatic SBRT has been on patients with LAPC, increasing evidence supports the potential benefits of SBRT for patients with BRPC. Again, advantages over conventional fractionated radiotherapy are the shortened treatment duration, which not only enhances patient comfort but also facilitates the integration with chemotherapy regimens, and the possibility of focused irradiation with sparing of adjacent structures.

Our search identified nine prospective studies published between 2016 and 2022, consisting of two observational studies, three phase I and three phase II studies (see Table 3). Patient numbers ranged from twelve to 45. However, the randomized phase II trial Alliance A021501 included 126 patients. Fractionation ranged from 30 Gy to 50 Gy in 3–5 fractions. Half of the studies included in our analysis applied a SIB to tumor-vessel-interface. Most studies applied SBRT after induction chemotherapy with mFOLFIRINOX. The resection rate ranged between 41 % and 80 % with R0 rates between 74 % and 100 %. The median OS ranged between 8 and 25 months. The variability between studies can be explained by differences in the intensity of induction chemotherapy, differences in staging imaging modalities (which may have resulted in an underestimation of the disease burden) and differences in the applied SBRT doses, which ranged from BED_10_ of ≈60 Gy [88] to ≈100 Gy [89].

**Table 3:** Prospective studies using SBRT for treatment of BRPC/RPC.

Study, Year	Study Design	Patients	Dose (Gy)	Fractions	Chemotherapy	Median OS (mos.)	Median PFS (mos.)	Resection Rate (%)	R0 Rate	Local failure	Toxicity
Shaib et al. 2016 [92]	Phase I	13 BRPC	36 to45 Gy: (25 % 30 Gy + 6 Gy SIB, 25 % 36 Gy + 6 Gy SIB, 25 % 36 Gy + 7,5 Gy SIB, 25 % 36 Gy + 9 Gy SIB)	3	4 cycles of induction mFOLFIRINOX (92 %)	All: 11 mos. (from enrolment), Resected patients: not reached in 18 mos. mFU	All: 6 mos. (from enrolment), Resected: not reached in 18 mos. mFU	61 %	95 %	63 % at 18 mos. mFU	No G 3/4 toxicities
Kharofa et al. 2019 [91]	Phase II	18 (15 BRPC / 3 RPC)	33 Gy GTV (optional25 Gy elective ENI 25	5	3 cycles of induction GEM/nab-paclitaxel (72 %) OR FOLFIRINOX (28 %)	All: 21 mos. (from enrollment), Resected: 31 mos., Non– resected: 9 mos.	All: 11 mos. Resected patients: 14 mos.	67 %	92 %	50 % at 12 mos. after OP	No G 3/4 or GI toxicity
Lin et al. 2019 [54]	Phase I	39 (22 BRPC/17LAPC)	25 to 40 Gy: (72 % 35–40 Gy/5 fx)	5	Induction CTX GEM/leucovorin/fluorouracil and concurrent nelfinavir	14 mos.	11 mos.	All: 31 % BRPC:41 % LAPC:17 %	85 %	15 %	10 % G 3, 5 % G 4 event, 13 % late GI
Chen-Zhao et al. 2020 [89]	Observational	45 (25 BRPC/ 5 RPC/ 15 LAPC)	40 to 62 Gy: (80 % 50 Gy/5fx, 2.2 % 62 Gy/10fx, 4.4 % 60 Gy/10fx, 11.1 % 50 Gy/10fx, 2.2 % 40 Gy/10fx)	5 (80 %) 10 (20 %)	3 cycles of induction FOLFIRINOX (13 %) or Gemcitabine/nab-paclitaxel (80 %) or XELOX (5 %), other (2 %)	21.8 mos. (1-year OS 68 %, 2-year OS 37 %)	14 mos. (1-year PFS 73 %, 2-year PFS 8 %)	71 %	94 %	5 % at 15 mos. mFU	No G 3/4 toxicities
Quan et al. 2020 [93]	Phase II	35 (19 BRPC/16LAPC)	36 Gy	3	Induction GEM/Cabacitabine 3 cycles (91 %)	All: 19 mos., BRPC: 28mos., LAPC: 14 mos.	Resected: 1-year LPFS 80 % Non-resected: 44 %	All 34 % BRPC: 53 %, LAPC: 13 %	92 %	BRPC: 36 %, LAPC: 78 %	No G 3/4 toxicities
Witt et al. 2021 [88]	Phase I	17 RPC	25–35 Gy (47 % 35 Gy) + ENI 25 Gy	5	concurrent capecitabine	NR	NR	75 %	100 %	23 %	56 % G2 nausea
Holyoake et al. 2021 SPARC[134]	Phase I	12 BRPC	30 to 32.5 Gy primary PTV 45 Gy-47.5 Gy boost volume (PTV_R)	5	Induction FOLFIRINOX (42 %)	All: 8 mos.	2 mos.	18 % (2Pt.)	50 % (1Pt.)	50 %	58 % G3 16 % G 4
Bouchart et al. 2022 [55]	Observational	39 (21 BRP/18 LAPC)	35 to 40 Gy primary PTV 53 Gy SIB to TVI	5	mFOLIRINOX (median 6 cycles)	All: 25 mos. resected 32 mos., non-resected 18 mos.	16mos. Resected: 24 mos., Non-resected: 7 mos.	56 % BRPC:72 % LAPC: 38 %	73.7 %	34 % at 18 mos. mFU	3 % acute GI 4 % late G3 GI
Katz et al. 2022 Alliance A021501 [104]	Phase II	126 BRPC	25 to 33 Gy (87.5 % 33 Gy, SIB up to 40 Gy, 12.5 % 25 Gy)	5	Induction mFOLFIRINOX (7 cycles)	Arm A 30 mos. Arm B 17 mos.	EFS: Arm A 15 mos. Arm B: 10 mos.	Arm A: 58 % Arm B: 51 %	Arm A: 88 % Arm B:74 % for arm B	NR	NR

**Abbreviations:** BRPC: borderline resectable pancreatic cancer; CTx: Chemotherapy; DLT: Dose limiting toxicity; Dx: Diagnosis; ENI: elective nodal irradiation; EFS: Event-free survival; FOLFIRINOX: oxaliplatin + folinic acid + irinotecan + fluorouracil; G = Grade; FFLP: Freedom from local progression; GI: Gastrointestinal; Gy: Gray; LAPC: Locally advanced pancreatic cancer; LC: Local control; LPFS: Local progression-free survival; mFu: median Follow-up; NR: not reported; OS: Overall survival; PFS: Progression free-survival; R0: negative resection; RPC: resectable pancreatic cancer; RT: Radiotherapy; SBRT: Stereotactic body radiotherapy; TVI: Tumor vessel interface.

Consistent with retrospective studies [62], [90], resected patients had significantly improved median OS compared to non-resected patients in several prospective studies [55], [91], [92], [93]. Reported local failure rates ranged between 15 % and 63 %. Rates of ≥ grade 3 toxicity ranged between 0 % and 10 %.

Furthermore, for elderly patients with medically inoperable PDAC, SBRT is emerging as a beneficial alternative to the commonly offered palliative chemotherapy or best supportive care [94], offering not only a low toxicity and promising local control rates [95], [96], [97], [98], [99], [100], [101], [102]. Additionally, the utilizing of MRgRT could further benefit elderly patients with medically inoperable PDAC [39], [47], [103]. Despite promising results, the majority of these findings are retrospective, underscoring the need for more robust, prospective studies to confirm these benefits.

The Alliance trial, previously mentioned, warrants detailed discussion. It randomized 126 patients to receive neoadjuvant mFOLFIRINOX (8 cycles, Arm A) or mFOLFIRINOX + SBRT (7 cycles, mainly 33–40 Gy in 5 fractions, Arm B). The primary objective was to compare 18-month OS with historical data, involving a separate assessment for the two treatment arms, and a comparison between arms only if both were deemed promising by a “pick a winner” strategy. The SBRT arm was halted prematurely following an interim analysis of the first 30 patients, as the rate of R0 resections (30 %) fell below a predefined threshold. Median OS was 30 months in arm A and 17 months in arm B. Toxicity ≥ grade 3 was 57 % in arm A and 64 % in arm B [104].

However, the trail raises notable concerns. Firstly, employing the R0 resection rate as a stopping criterion is problematic due to the lack of re-staging at the end of chemotherapy. Early progression events likely contributed more to the lower resection rate in arm B than the effects of local radiotherapy. This is also supported by the markedly lower resection rate, compared to prior studies [15], [85]. Indeed, there was a higher rate of pre-surgery metastasis in arm B, which can be attributed to the omission of one chemotherapy cycle or underlying unfavorable biological factors. Furthermore, the study's stratification was limited to performance status, neglecting other potential influencing factors. Inter-arm differences, including treatment delays and chemotherapy dose reductions, further complicate the analysis. Moreover, criticisms include suboptimal radiation application, such as low BED_10_ values for some patients and lack of reported vascular boosts at tumor-vessel-interfaces. Lastly, the study's pick-the-winner design does not allow for comparisons of primary and secondary end points between the selection arms after arm B was discontinued [105].

These concerns highlight the need for further research to determine the true impact of SBRT on outcomes in patients with RPC or BRPC. Although there are no data in particular, it can be assumed by analogy with LAPC that further dose escalation is also useful in BRPC, since PADC is known to have low radiation sensitivity [66]. In addition, criteria besides vascular involvement needs to be found for selecting those patients who will benefit from local therapies.

### 3.4 SBRT as an adjuvant treatment for patient with resected pancreatic cancer

Surgery followed by adjuvant chemotherapy is the standard of care in RPC and achieve a 5-year OS > 40 % [16], [106], [22]. Nevertheless, the prognosis following positive margin resections remains poor, providing the rationale for adjuvant local therapy strategies [78]. However, the role of adjuvant radiotherapy is debatable: Several randomized trials on adjuvant CRT led to conflicting results and were criticized for outdated treatment techniques [107], [108], [109], [110].

Our search revealed two prospective trials for SBRT (Table 4). The prospective observational study by Bernard demonstrated the feasibility of adjuvant SBRT with 36 Gy in 3 fractions in patients with positive or close margins [111]. Most patients received neoadjuvant and adjuvant gemcitabine-chemotherapy. The study found a median OS of 24 months, which is comparable with the results of trials utilizing adjuvant chemotherapy with gemcitabine alone. However, LC rates at one and two years of 85 % and 77 %, respectively, compared favorably. Acute and late ≥ grade 3 toxicity was mild with 4.1 % and 0 %, respectively.

**Table 4:** Prospective studies using SBRT as an adjuvant approach in pancreatic cancer.

Study, Year	Phase	Patients	Stage	Resection status	Dose (Gy)	Fractions	CTx	Median OS (mos.)	LRFS	Toxicity
Bernard et al. 2018 [111]	Prospective observational	49 (With positive/close margin)	RPC 55 %, BRPC 43 %. LAPC 2 %	R1, 78 %, R0 22 %	36	3	Neoadjuvant 65 %, Adjuvant 82 %	All: 20 mos. R1: 16 mos. R0: 22 mos.	1 yr.: 85 % 2 yr.:77 %,	4 % acute G3 no late G 3 + toxicity
Ma et al. 2022 [112]	Prospective randomized	38 20-> GEM 18-> GEM + SBRT	Resected Stage II PDAC	R0 100 %	25	5	Concurrent GEM	GEM- Arm: 28 mos. GEM + SBRT: 15 mos.	unreached in both arms	NR in details, comparable G 3 or 4 toxicity between the two arms.

**Abbreviations:** BRPC: borderline resectable pancreatic cancer; CTx: Chemotherapy; FFLP: Freedom from local progression; G = Grade; Gy: Gray; LAPC: Locally advanced pancreatic cancer; LC: Local control; LRFS: Locoregional Recurrence-Free Survival;RPC: resectable pancreatic cancer; NR: not reported; OS: Overall survival; PDAC: Pancreatic Adenocarcinoma; R0: negative resection; R1: positive resection; RT: Radiotherapy; SBRT: Stereotactic body radiotherapy.

The second study by Ma is a prospective randomized single-center trial, comparing adjuvant SBRT with 25 Gy in 5 fractions and concurrent gemcitabine to gemcitabine alone [112]. However, adjuvant SBRT did not yield any advantage over gemcitabine alone in terms of LC and OS.

In summary, although available prospective data show no survival benefit over chemotherapy alone, SBRT might improve LC in patients with positive resection margins. However, the evidence is low, and the approach is challenged by more aggressive neoadjuvant treatments. Further prospective trials are warranted before SBRT can be recommended in the adjuvant setting.

### 3.5 SBRT as salvage option after local recurrence in pancreatic cancer

Relapse occurs in 70 % − 80 % of patients after surgical resection [16], with ILR occurring in 17 % − 30 % [113], [114]. However, there is no established standard of care for this clinical situation. With curative re-resection a median OS up to 26 months is achieved in retrospective series, but usually, local recurrent disease is unresectable [115]. Few retrospective studies show that conventional CRT is feasible for ILR with median OS between 10 and 17 months [116], [117], [118]. However, the possibility of dose-escalation while sparing surrounding OAR in few fractions makes SBRT attractive. Moreover, patients experiencing local recurrence frequently suffer from cancer-related abdominal pain. SBRT can alleviate these symptoms and enhance overall quality of life.

We identified two prospective studies investigating SBRT for ILR (Table 5). The first is a prospective observational study conducted by Li to evaluate SBRT with median 40 Gy in four to seven fractions using Cyber Knife. They reported a median OS of 11 months and a LC of 82 % and 37 % at 6 and 12 months, respectively. Symptom alleviation was observed in 16 of 17 patients (94 %) within 2 weeks after SBRT. Toxicity was mild with only 4 % ≥ grade 3 late toxicity [119].

**Table 5:** Prospective studies using SBRT for treatment ILR.

Study, Year	Phase	Intervention	Patients	Dose (Gy)	Fractions	Systemic therapy	Median OS (mos.)	Toxicity	Local control
Li et al. 2020 [119]	Prospective observational	SBRT using Cyber Knife (CK) in patients with recurrent pancreatic cancer	27 patients	Median 40 Gy (range 25–50 Gy)	4 to 7	67 % sequential CTx	11 mos.	78 % acute G 1–2, 4 % late G 3 toxicity	37 %
Zhu et al. 2021 [120]	Phase II	SBRT plus pembrolizumab and trametinib in patients with recurrent pancreatic cancer	170 patients. Arm A (n = 85): SBRT plus pembrolizumab and trametinib Arm B (n = 85): SBRT plus gemcitabine	35 to 40 Gy	5	Pembrolizumab and trametinib (50 %) Gemcitabine (50 %)	Arm A: 25 mos. Arm B: 22 mos.	No G3 + GI Mostly drug related toxicity	NR

**Abbreviations:** G = Grade; Gy = Gray; NR: not reported; OS: Overall survival; CK: Cyber knife; ILR: isolated local recurrence; RT: Radiotherapy; SBRT: Stereotactic body radiotherapy.

The second study, a recent phase II study by Zhu, compared the efficacy of stereotactic body radiotherapy with 35–40 Gy in 5 fractions combined with pembrolizumab and trametinib versus SBRT plus gemcitabine in 170 patients. The study found an impressive median OS of 25 months from randomization for patients treated with SBRT, pembrolizumab and trametinib, compared to 22 months for those treated with SBRT and gemcitabine. Toxicity consisted mainly of drug-induced adverse events. However, since the trial focused on different systemic therapies, not much information about the radiation treatment was provided [120].

In light of the fact that prospective literature on SBRT for ILR is sparse, the following retrospective studies should be mentioned: Comito reported on 31 patients treated with 45 Gy in 6 fractions. Median OS was 18 Gy and LC was 91 % and 82 % at one and two years, respectively. No cases of acute G3 toxicity or greater occurred [121].

In addition, SBRT presents an option in the context of re-irradiation. Dagoglu retrospectively evaluated SBRT re-irradiation in 30 patients. Among these, 20 had undergone conventional fractionated treatment and ten had received SBRT. The median re-irradiation dose was 25 Gy in 5 fractions. The median OS was 14 months and LC rates at one and two years were 78 %. Acute and late grade 3 toxicity was 11 % and 7 %, respectively [122].

Sutera et al., reported on 38 patients undergoing salvage SBRT re-irradiation with a median dose of 24.5 Gy in 1–3 fractions after previous conventional radiotherapy. The median OS from initial diagnosis was 27 months and 10 months from SBRT re-irradiation. Late ≥ grade 2 and ≥ grade 3 toxicities were 18 % and 10 %, respectively [123].

### 3.6 SBRT for pain relief as a palliation option

Pain emerges as the primary symptom in about 30 %-40 % of PDAC patients upon diagnosis and becomes even more prevalent before death, affecting up to 90 % [124]. For patients with an inadequate response to pain medications, there are several local procedures that may provide temporary relief from symptoms [125], [126], including celiac plexus block, radiofrequency ablation, irreversible electroporation, and high intensity focused ultrasound. Among these options, radiotherapy stands out for its non-invasive nature and positive impact on quality of life [124], [127], [128], [129].

However, in a palliative setting with limited patient lifespan, short treatment times and the best possible avoidance of side effects are paramount, making SBRT attractive in comparison with conventional radiotherapy.[130] Retrospective data show that SBRT offers long-lasting pain reduction in approximately two-thirds of patients experiencing symptoms [128], [129].

Our search revealed one prospective phase II trial investigating palliative SBRT with pain severity reduction as primary endpoint. The application of 24 Gy in three weekly fractions led to a significant pain reduction in 80 % of patients, a reduction in pain medication in 55 % and to improved quality of life scores, within an acceptable toxicity profile [131].

Moreover, another five studies included in our analysis reported on pain relief after SBRT, although not as primary end point. In the study by Hermann et al., stereotactic body radiotherapy was found to significantly decrease the pancreatic pain score as measured by the QLQ-PAN26 questionnaire after four weeks of treatment, with a favorable toxicity profile [33]. Gurka et al. could not show a significant improvement in symptoms using the same questionnaire. Nonetheless, there was a trend towards improved back pain, night pain and abdominal discomfort [37]. Liauw reported a pain response in 63 % of LAPC patients with pre-existing pain utilizing a numeric pain rating scale (NRS) before and after SBRT [35]. In the study of Tozzi, pain relief on NRS was observed in 100 % of patients with pre-existing pain. Analgesics could be suspended in 64 % and reduced by 50 % in another 27 % of patients [38]. Furthermore, Doppenberg reported pain relief in 30 of 36 patients (83 %) [44]. These results from prospective studies correspond very well to the findings of two systematic reviews reporting a pain relief rate of 85 % [128], [129].

Another notable, albeit retrospective, study examined short-course palliative SBRT using single-fraction (median 25 Gy) and five-fractions regimens (median 33 Gy). Interestingly, single-fraction SBRT yielded significantly better pain relief (64 % versus 10 %)[132].

In summary, there is existing evidence supporting the potential of SBRT for effective and long-lasting pain control in primary pancreatic cancer. However, further research through prospective randomized trials is necessary to validate these findings and to find the optimal dose needed. One ongoing international multicenter trial, known as MASPAC, aims to examine the efficacy of MR-guided SBRT in providing pain relief for patients with metastatic pancreatic cancer who have a stable disease after initial systemic treatment[133].

### 3.7 Limitations

The present review has several limitations: (1) Limited high-quality evidence: Although all included studies are prospective, they primarily consist of single-institution studies, lacking multicenter randomized controlled trials. (2) Prospective focus: Our emphasis on prospective data may have led to some oversight of relevant findings from retrospective studies, however, we have attempted to include pertinent retrospective data when warranted. (3) Small sample sizes: A number of studies included in the review had relatively small patient cohorts. (4) Heterogeneity in parameters: Variability in the definition of planning target volumes, doses, fractionation schemes, delivery systems, and image guidance techniques made direct comparability difficult. (5) Missing critical data: Some of the included studies lacked essential information, such as LC or resection rates. However, we believe that our systematic approach in gathering the most current prospective evidence offers distinct advantages compared to recent reviews, which heavily rely on retrospective data.

## 4 Conclusion

Prospective data on SBRT in various PDAC clinical scenarios are emerging. SBRT demonstrates promising outcomes with good LC rates and, in some cases, substantial resection rates in LAPC. The use of MRgRT may provide a solution to the challenge of delivering ablative doses while mitigating severe toxicities. However, whether this approach confers additional survival benefits compared to multi-agent chemotherapy alone requires validation through randomized trials. Despite the results of the Alliance Trial, neoadjuvant SBRT should not be dismissed outright. Single prospective studies combining preoperative ablative dose SBRT with modern induction systemic therapies have achieved impressive resection rates of up to 80 %. MRgRT also holds potential in this context, and ongoing randomized trials will probably identify the best neoadjuvant strategy within the next years. Current evidence doesn’t demonstrate a survival advantage of SBRT as adjuvant therapy over chemotherapy alone, However it might improve local control in patients with positive resection margins following radical resection. Prospective data for SBRT in ILR and for pain relief are limited; however, they seem to confirm the positive retrospective results. In general, there is a demand for randomized controlled trials in well-defined patient cohorts to improve our understanding of SBRT's clinical applications in various PDAC scenarios.

## Declaration of competing interest

The authors declare that they have no known competing financial interests or personal relationships that could have appeared to influence the work reported in this paper.

## Appendix Search Terms

((pancreatic OR pancreas) AND (Cancer OR Carcinoma OR adenocarcinoma OR PDAC)) AND ((stereotactic body radiation therapy) OR (stereotactic body radiation) OR SBRT OR (magnetic resonance guidance radiation therapy) OR (MRgRT) OR (MR Linac)).
